# Crosstalk between Beta-Catenin and Snail in the Induction of Epithelial to Mesenchymal Transition in Hepatocarcinoma: Role of the ERK1/2 Pathway

**DOI:** 10.3390/ijms141020768

**Published:** 2013-10-16

**Authors:** Nathalie Zucchini-Pascal, Ludovic Peyre, Roger Rahmani

**Affiliations:** Laboratory of Xenobiotic’s Cellular and Molecular Toxicology, INRA, UMR 1331 TOXALIM (Research Centre in Food Toxicology), Sophia Antipolis 06903, France; E-Mails: zucchini@sophia.inra.fr (N.Z.-P.); rahmani@sophia.inra.fr (R.R.)

**Keywords:** epithelial-mesenchymal transition, ERK1/2, hepatocellular carcinoma, β-catenin, snail

## Abstract

Epithelial to mesenchymal transition (EMT) is an integral process in the progression of many epithelial tumors. It involves a coordinated series of events, leading to the loss of epithelial features and the acquisition of a mesenchymal phenotype, resulting in invasion and metastasis. The EMT of hepatocellular carcinoma (HCC) cells is thought to be a key event in intrahepatic dissemination and distal metastasis. In this study, we used 12-*O*-tet-radecanoylphorbol-13-acetate (TPA) to dissect the signaling pathways involved in the EMT of HepG2 hepatocarcinoma cells. The spectacular change in phenotype induced by TPA, leading to a pronounced spindle-shaped fibroblast-like cell morphology, required ERK1/2 activation. This ERK1/2-dependent EMT process was characterized by a loss of E-cadherin function, modification of the cytoskeleton, the acquisition of mesenchymal markers and profound changes to extracellular matrix composition and mobility. Snail was essential for E-cadherin repression, but was not sufficient for full commitment of the TPA-triggered EMT. We found that TPA triggered the formation of a complex between Snail and β-catenin that activated the Wnt pathway. This study thus provides the first evidence for the existence of a complex network governed by the ERK1/2 signaling pathway, converging on the coregulation of Snail and the Wnt/β-catenin pathway and responsible for the onset and the progression of EMT in hepatocellular carcinoma cells.

## 1. Introduction

Hepatocellular carcinoma (HCC) is the fifth most commonly diagnosed, and the third most deadly, cancer worldwide [1,2]. Hepatocarcinogenesis is a multistep process, slowly unfolding against a backdrop of chronic liver disease, including chronic hepatitis and cirrhosis, which are regarded as preneoplastic [3–5]. The early steps of HCC development are influenced by both epigenetic and genetic mechanisms [4,6]. In later stages, the escape of carcinoma cells from the solid tumor may result from the dedifferentiation of epithelial cells, through a loss of cell-to-cell contact accompanied by an increase in migratory and invasive capacities [7]. This phenotypic conversion, collectively referred to as the epithelial to mesenchymal transition (EMT), is considered to constitute an important process in intrahepatic dissemination and distal metastasis during HCC progression [8,9].

EMT plays a key role in the critical phases of embryonic development and contributes to both physiological processes, such as tissue repair, and pathological conditions, including carcinogenesis and fibrosis, in adults [10]. It allows a polarized epithelial cell to undergo multiple biochemical changes leading to the acquisition of a mesenchymal cell phenotype. EMT occurs as a sequence of steps, beginning with the loss of apico-basal polarity, due to dissolution of the tight junctions, resulting in the intermingling of apical and basolateral membrane components [8,11,12]. Additional cell-to-cell junctions (adherens and gap junctions) are disrupted and the underlying basement membrane is broken down [13]. Cell surface proteins, such as E-cadherin and integrins, responsible for connecting epithelial cells to neighboring cells and to the basement membrane, respectively, are replaced by *N*-cadherin and integrins conferring more transient adhesive properties on the cell, priming it for adoption of the mesenchymal phenotype [14]. The cytoskeleton is also reorganized, to enable the cell to move through the three-dimensional extracellular matrix (ECM). In particular, the structure of the actin cytoskeleton undergoes dynamic changes, from a cortical actin network to stress fibers in areas of cell protusion. The epithelial cytokeratin intermediate filaments are replaced by vimentin [15]. Together, these changes cause a transition in cell shape from cuboidal to spindle-shaped. Finally, the cell acquires a motile and invasive phenotype.

These morphological changes are governed by multiple molecular mechanisms, including the loss of E-cadherin function, a crucial step in EMT [14,16]. Low levels of E-cadherin have been observed in HCC and are associated with a poor prognosis [17], reflecting the critical role of the loss of this protein in tumor progression. E-cadherin is thought to be downregulated via several repressors acting either indirectly (e.g., Twist, Goosecoid) or directly, by binding to and repressing the E-cadherin promoter (e.g., Snail, Slug, Zeb) [18,19].

E-cadherin repression is frequently accompanied by activation of the β-catenin/Wnt signaling cascade [20]. β-catenin, a member of the protein complex connecting cadherins to the actin cytoskeleton at adherens junctions, plays a crucial role in the onset and progression of EMT. In normal unstimulated cells (*i.e.*, in the absence of the Wnt signal), β-catenin levels are regulated by a multiprotein complex consisting of adenomatous polyposis coli (APC) tumor suppressor protein, axin, casein kinase 1 (CK1) and the glycogen synthase kinase, GSK3β. In steady-state conditions, the cytoplasmic β-catenin is thus bound to this complex, leading to its phosphorylation by casein CK1 and GSK3-β at specific serine/threonine residues. This sequential phosphorylation targets β-catenin for ubiquitination and ultimate degradation by the proteasome [21,22]. Docking of the Wnt ligand to its Frizzled (Fz) receptor triggers activation of the canonical Wnt pathway, in a cascade of events that destabilizes the degradation complex, allowing unphosphorylated β-catenin to accumulate and to be translocated to the nucleus. Within the nucleus, β-catenin binds to lymphoid-enhancing factor/T-cell factor (LEF/TCF), to initiate the transcription of target genes, such as TCF1 (transcription factor 1), CD44, MMP-7 (matrix metallo-proteinase-7) and cyclin D1 [23–26].

The EMT of neoplastic hepatocytes is thought to be a key event in metastasis. We therefore investigated the events underlying the EMT of hepatoma cells. We used the tumor promoter 12-*O*-tet-radecanoylphorbol-13-acetate (TPA), which has been reported to induce EMT in differentiated HCC cells derived from the HepG2 cell line [27]. We found that TPA-triggered ERK1/2 activation played a critical role in the onset and progression of EMT. We also found that EMT in HepG2 cells resulted from co-activation of the Snail transcription factor and the Wnt/β-catenin signaling pathway, amplifying EMT progression. Our data also reveal the existence of an intriguing network, governed by the ERK1/2 signaling pathway and responsible for regulating EMT in the dedifferentiation of hepatocellular carcinoma cells.

## 2. Results and Discussion

### 2.1. ERK1/2 Activation Is Critical for TPA-Mediated Hepatoblastoma Cell Migration

**Figure 1:**
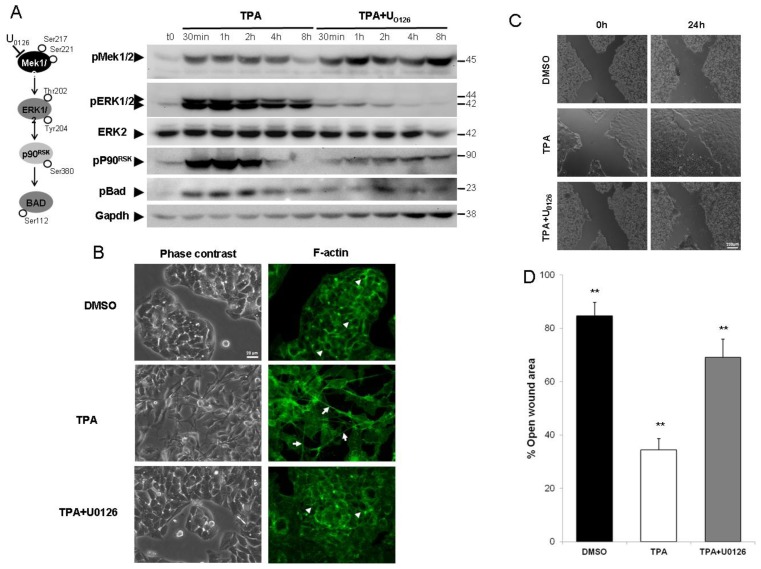
The inhibition of ERK1/2 activation by U0126 prevents HepG2 cell migration. (**A**) HepG2 cells were exposed to 100 nM tumor promoter 12-*O*-tet-radecanoylphorbol-13-acetate (TPA) for 30 min to 4 h, with or without U0126. Cells were lysed and subjected to western blotting, as described in Section 2. The ERK1/2 signaling pathway was studied by analyzing the phosphorylation of MEK1/2, ERK1/2, P90rsk and Bad, together with ERK2 levels. As a control, the same membranes were also probed with an antibody directed against Gapdh; (**B**) 24 h after TPA treatment, cell morphology was examined under a light microscope. F-actin was visualized by AlexaFluor 488-conjugated phalloidin staining (green) and fluorescence microscopy; (**C**,**D**) The subconfluent HepG2 cells were wounded 48 h after plating and exposed to TPA or TPA + U0126. Images were obtained 24 h after treatment (**C**). Percentages of open wound area at 24 h, in each set of conditions, as in (**C**), were plotted, with wound width was normalized with respect to the initial value at 0 h (**D**). Errors bars indicate the mean ± SEM of triplicate determinations in three independent experiments. ** *p <* 0.01.

The activation of MAPK pathways (including ERK, p38 and JNK) by TPA treatment has been reported in several cell models. Hence, we studied the kinetics of MAPK activation in HepG2 cells by western blotting with antibodies directed against phosphorylated forms of ERK1/2, p38 (data not shown) and JNK (data not shown), to analyze kinase activation. The ERK1/2 signaling pathway was the most rapidly and strongly activated of the MAPK pathways tested. Indeed, TPA treatment led to a considerable increase in the amounts of phosphorylated forms of ERK1/2 (pERK1/2) and their known downstream effectors (Figure 1A). This activation occurred within five minutes of TPA treatment (data not shown) and peaked after about one hour. As expected, the use of U0126, a specific inhibitor of MEK1/2, abolished all downstream ERK1/2 signal transduction (Figure 1A).

Because TPA has been reported to induce EMT-like cell scattering in HepG2 cells [27], we used U0126 to determine whether ERK1/2 activation was responsible for this process. In control conditions, HepG2 cells had an epithelial cell-like morphology with a characteristic “cobblestone” appearance and the organization of F-actin into a cortical pattern at cell-to-cell junctions (Figure 1B, arrowhead). Phase contrast microscopy revealed that the cells underwent a change in morphology in response to TPA treatment, acquiring a pronounced spindle-shaped fibroblast-like cell morphology within 24 h of treatment, with polarization of the F-actin stress fibers throughout the cell (Figure 1B, arrows). HepG2 cells treated with TPA in the presence of the MEK1/2 inhibitor U0126, underwent little or no change in morphology, contrasting strongly with the results obtained when the cells were treated with TPA alone (Figure 1B). U0126 had no apparent effect on cell morphology (data not shown). We investigated the possible functional impact of ERK1/2 pathway blockade on TPA-induced cell migration, by carrying out wound-healing assays. TPA treatment resulted in more complete and rapid wound closure (Figure 1C,D) than the control treatment (DMSO). U0126 slowed TPA-mediated cell migration by a mean of 70% (*p* < 0.01), while it had no effect on the basal wound closure (data not shown).

These results revealed that ERK1/2 activity is directly involved in the morphological transformation and cell migration induced by TPA.

### 2.2. Inhibition of the ERK1/2 Pathway Abrogates the TPA-Mediated Deregulation of Epithelial and Mesenchymal Markers

Cell migration is often considered a hallmark of EMT. We therefore investigated whether the TPA-induced ERK1/2-dependent migration of cells was consistent with EMT. This process is classically associated with the deregulation of defined molecular markers, including cell-surface, cytoskeletal and extracellular proteins.

**Figure 2:**
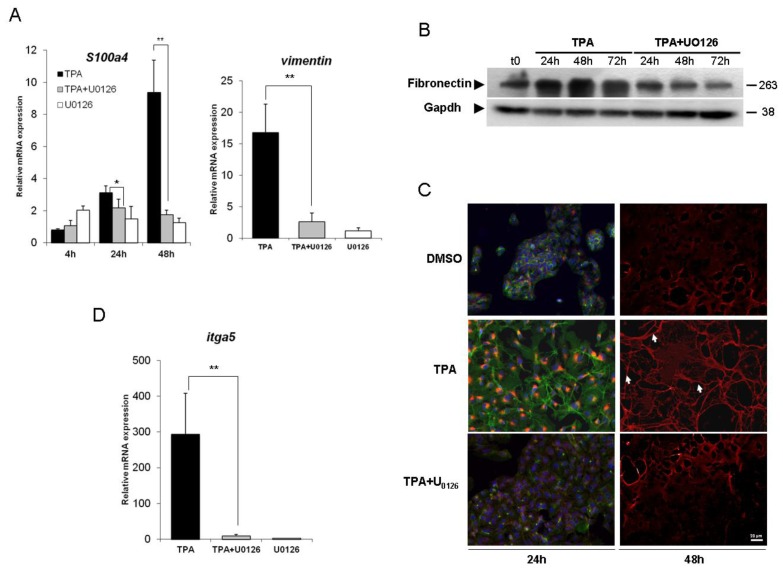
ERK1/2 inhibition reverses the epithelial to mesenchymal transition (EMT) process induced by TPA. (**A**) changes in mRNA levels for the EMT-related *S100a4* and *vimentin* genes were assessed by real-time RT-PCR. The HepG2 cells were stimulated with 100 nM TPA, with or without U0126, for 4 h, 24 h and 48 h (*S100a4*) or 48 h (v*imentin*); (**B**) Cells were exposed to 100 nM TPA, with or without U0126, for 24 to 72 h. At the indicated times, cells were lysed and fibronectin protein levels were assessed by western blotting (results representative of three experiments); (**C**) HepG2 cells were grown on coverslips and treated with TPA with or without U0126 for 24 h (intracellular fibronectin) or 48 h (fibronectin deposition). After exposure, the cells were fixed and processed for indirect immunofluorescence analysis for the detection of fibronectin (red) and visualization of the actin cytoskeleton (phalloidin, green) and nuclei (DAPI, blue). The results shown are representative of three independent experiments; (**D**) itga5 mRNA levels were assessed by real-time RT-PCR after 48 h of TPA treatment, with or without U0126. For all real-time RT-PCR experiments (**A** and **D**), relative mRNA levels with respect to *gapdh* mRNA levels are given, and the mRNA levels in DMSO-treated cells are taken as 1. Errors bars indicate the mean ± SEM of triplicate determinations in three independent experiments. ** *p <* 0.01.

We first investigated the effect of TPA on the cytoskeletal markers S100a4 and vimentin. Levels of mRNA for S100a4, a marker of hepatocytes undergoing EMT [28], increased considerably in response to TPA treatment, in a concentration- and time-dependent manner. These levels had increased by a factor of about 10, 48 h after TPA and their increase was abolished by simultaneous treatment of the cells with U0126. Vimentin is an intermediate filament protein normally found in cells of mesenchymal origin in physiological conditions or in migrating epithelial cells. The presence of this protein is commonly used to identify cells undergoing EMT in cancers [29,30]. In HepG2 cells, the vimentin gene was weakly expressed in basal conditions (data not shown). However, after 48 h of TPA treatment, vimentin mRNA levels had considerably increased (Figure 2A). Note that no significant transcriptional induction was observed upon TPA exposure before 48 h (data not shown). This effect was ERK1/2-dependent, because it was abolished by U0126 treatment.

Extracellular matrix (ECM) remodeling is another major phenotypic modification observed during EMT. Fibronectin, a high-molecular weight glycoprotein, serves as a scaffold for the fibrillar ECM and is one of the first molecules to appear during the formation of the fibrillar ECM. We therefore analyzed fibronectin levels by western blotting and immunofluorescence studies. Western blot analysis (Figure 2B) showed that TPA treatment induced a time-dependent upregulation of fibronectin protein levels over control conditions (t0), and that this effect was prevented by U0126. These findings are consistent with the synthesis of large amounts of fibronectin in the cytoplasm of HepG2 cells detected by immunofluorescence analysis after 24 h of TPA treatment (Figure 2C). Fibronectin was deposited as fibrils (arrows) in the extracellular compartment, 48 h after TPA exposure. Interestingly, this extensive fibronectin matrix was significantly decreased by exposure to U0126. The formation of fibronectin fibrils requires the presence of fibronectin receptors, such as α5β1 [31]. As for vimentin, α5 transcripts (itga5) were weakly expressed in untreated condition (data not shown). The overproduction of fibronectin was accompanied by a significant induction of this gene in cells treated for 48 h with TPA (Figure 2D). In the presence of U0126, this effect was abrogated. It is to note that U0126 had no effect on the expression and the localization of fibronectin (data not shown).

Thus, HepG2 cells exposed to TPA underwent an EMT process in an ERK1/2-dependent fashion.

### 2.3. ERK1/2 Is Critical for TPA-Mediated *CDH1* Repression

**Figure 3:**
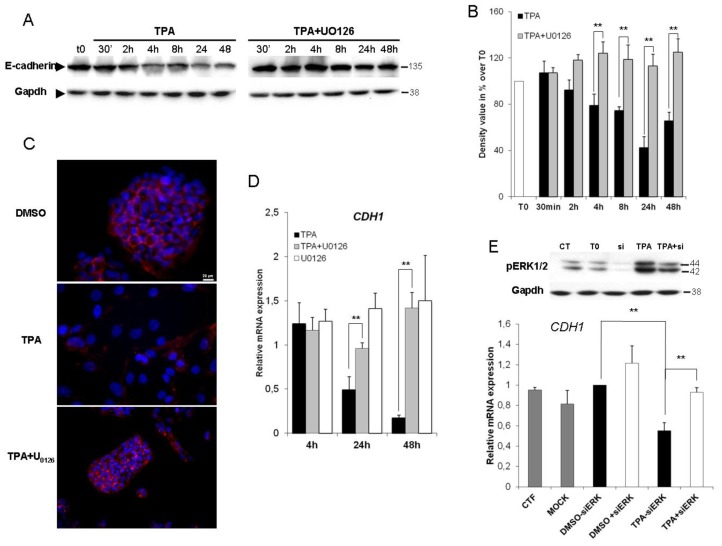
The repression of E-cadherin in TPA-exposed HepG2 cells is ERK1/2-dependent. HepG2 cells were treated with 100 nM TPA in the presence or absence of U0126. (**A**) At the indicated time, cells were lysed and E-cadherin protein levels were assessed by western blotting; (**B**) Band intensities were assessed by densitometry after image acquisition with a CCD camera and the results are presented as the ratio of Gapdh-normalized results for treated cells to those for DMSO-treated cells (means ± SD for three experiments). ** *p <* 0.01; (**C**) HepG2 cells were grown on coverslips and treated with TPA with or without U0126 for 48 h. After exposure, the cells were fixed and processed for indirect immunofluorescence analysis for the detection of E-cadherin (red) and visualization of nuclei (DAPI, blue). The results shown are representative of three independent experiments; (**D**) E-cadherin (*CDH1*) mRNA levels were assessed by real-time RT-PCR after 4, 24 and 48 h of TPA treatment, with or without U0126. Relative mRNA expression levels (normalized with respect to *gapdh*) were determined and mRNA levels in DMSO-treated cells were set to 1. Error bars indicate the means ± SEM of triplicate determinations from three independent experiments. ** *p <* 0.01; (**E**) HepG2 cells were transfected by incubation with either 40 pmol Snail siRNAs or nonspecific siRNAs (CT) for 36 h. HepG2 siRNA transfectants were exposed to 100 nM TPA for 24 h. Total proteins were extracted and level of phosphorylated Erk1/2 was examined by western blotting. As a control, the same membranes were also probed with an antibody directed against Gapdh. *CDH1* mRNA levels were assessed by real-time RT-PCR. Relative mRNA expression levels (normalized with respect to *gapdh*) were determined and mRNA levels in DMSO-treated cells (untransfected condition) were set to 1. Error bars indicate the means ± SEM of triplicate determinations from three independent experiments. ** *p <* 0.01.

As E-cadherin downregulation is the prototypic hallmark of EMT, we then investigated E-cadherin protein levels in untreated (t0) and TPA-treated cells in the presence and absence of U0126. In HepG2 cells, exposure to TPA for 30 min to 48 h resulted in significantly lower levels of E-cadherin protein (≈60% at 24 h) than in control conditions, and this decrease was prevented by U0126 (Figure 3A,B). In the absence of TPA, U0126 had no apparent effect on E-cadherin protein levels (data not shown). Indirect immunofluorescence studies (Figure 3C) showed that E-cadherin was restricted to zones of cell-to-cell contact in control conditions. In the presence of TPA, its distribution was disorganized, resulting in a loss of membrane labeling. Co-treatment with U0126 prevented the effect of TPA. As expected, the expression of E-cadherin (*CDH1*) gene decreased within 24 h of TPA exposure, and a sustained decrease was observed thereafter (≈80% after 48 h of TPA treatment). Inhibition of ERK1/2 activity restored the basal level of *CDH1* expression. One the other hand, it has been described that TPA activated the c-Jun *N*-terminal protein kinases (JNKs) [32]. To ensure that effects observed using the MEK1/2 inhibitor U0126 were attributable to ERK1/2, we used specific small interfering RNAs (siRNAs) directed against ERK1/2. As shown in Figure 3E, the ERK1/2 silencing significantly improved the down-regulation of *CDH1* caused by TPA. TPA therefore seems to abolish the transcription of the *E-cadherin* gene in an ERK1/2-dependent manner.

**Figure 4:**
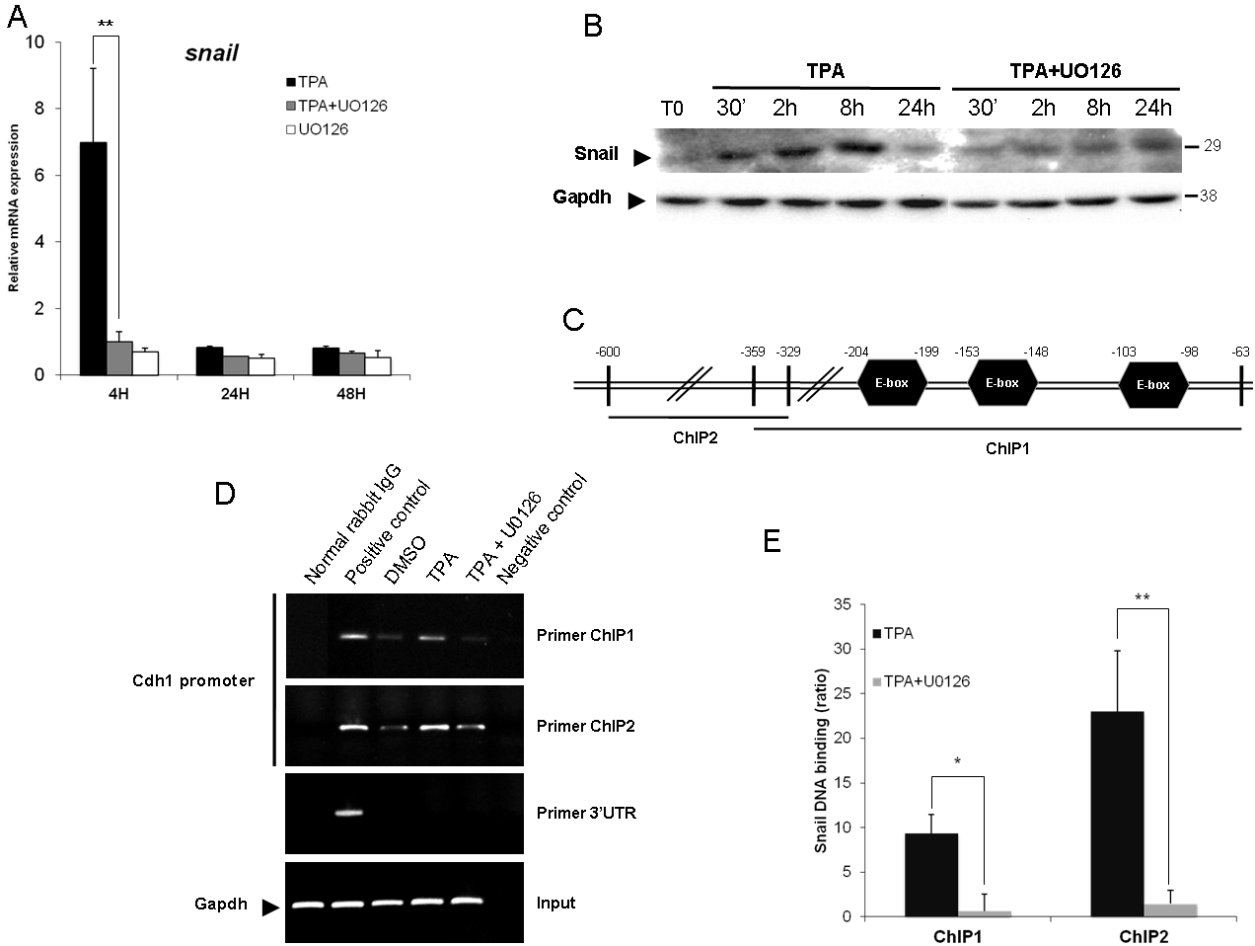
The binding of Snail to the E-box of the *CDH1* promoter requires ERK1/2 activation. (**A**) Levels of Snail mRNA were assessed by real-time RT-PCR. The HepG2 cells were stimulated with 100 nM TPA, with or without U0126, for 4 h, 24 h and 48 h. Relative mRNA expression levels (with respect to *gapdh*) were determined, with the mRNA levels in DMSO-treated cells set to 1; (**B**) Total proteins were extracted after 30 min, 2 h, 8 h and 24 h and the expression level of Snail was examined by western blotting. As a control, the same membranes were also probed with an antibody directed against Gapdh; (**C**) Diagram of the *CDH1* promoter showing the amplification site used for ChIP analyses; (**D**) HepG2 cells were exposed for 4 h to 100 nM TPA with or without U0126. Rabbit anti-Snail, normal rabbit IgG (negative control) and mouse anti-RNA Pol II (positive control) antibodies were used for immunoprecipitation. Input DNA, together with immunoprecipitated and purified DNA, was used for the amplification by PCR of a 296 bp (ChIP1) or a 271 bp (ChIP2) fragment specific to the *CDH1* promoter; (**E**) Band intensities were assessed by densitometry after image acquisition with a CCD camera and the results are presented as the ratio between the value for treated cells to that for DMSO-treated cells, both these values being normalized according to input (means ± SD for three experiments). The data presented are representative of three independent experiments. * *p <* 0.05; ** *p <* 0.01.

The *CDH1* promoter is frequently repressed, directly or indirectly, by specific transcriptional repressors, such as Snail, Slug and Twist 1. Previous study had shown that the EMT triggered by TPA is dependent on Snail [27]. Accordingly, we sought to determine if ERK1/2 was responsible for the Snail activation. Because this protein is extremely labile (half-life of only 25 min), we firstly used qPCR analysis to determine whether TPA directly affected Snail gene expression. mRNA levels were about seven times higher in HepG2 cells exposed to TPA than in control cells, and the TPA-induced expression of Snail was prevented by simultaneous incubation with U0126 (Figure 4A). This induction was both rapid (within 4 h of TPA treatment) and transient. It is to note that TPA treatment did not increase expression of the Twist, Slug or Zeb genes (data not shown), confirming the role of Snail as the most important E-cadherin repressor activated by TPA in HepG2 cells. Due to the instability of the Snail protein [33], we sought to determine if the up-regulation of Snail gene expression came with a sustained protein expression. As shown in Figure 4B, TPA upregulated Snail in a time dependent manner, as soon as 30 min when compared to T0 condition. It is to note that this protein increased was transient, as the Snail level diminished after 24 h TPA treatment. This increased Snail level observed during 24 h TPA treatment is prevented by the U0126.

Secondly, we investigated the requirement of the ERK1/2 signaling pathway for the E-cadherin repression induced by TPA, using chromatin immunoprecipitation (ChIP) assays. PCR-amplified fragments specific to the *CDH1* promoter region containing the E-box (obtained with two different primer pairs, ChIP1 and 2, Figure 4B) gave a weak signal for DNA extracted in control conditions (DMSO and U0126, data not shown) and immunoprecipitated with an antibody against Snail. The treatment of Hepg2 cells for 45 min with TPA resulted in significant binding of Snail to the E-cadherin promoter, and this effect was strongly decreased by simultaneous incubation with U0126 (Figure 4C,D). Thus, TPA induced the Snail binding on the *CDH1* promoter via an upregulation (Figure 4A,B) and an increased translocation of this transcription factor in the nucleus (Figure 4D,E). Indeed, as transcription factors, Snail proteins must translocate to the nucleus in order to be functional. The co-incubation with U0126 could diminish the *CDH1* promoter occupancy as the result of diminished Snail protein level and/or affinity of Snail for E-box sequences. However, the fact that U0126 prevented the upregulation of Snail at the mRNA and protein levels suggest that data obtained from ChIP experiments were due to the decreasing expression, nuclear translocation and functionality of Snail.

These results are consistent with previous findings and demonstrate that the binding of Snail to the overlapping E-box sequences in the E-cadherin promoter is dependent on ERK1/2 activity. Altogether, these data demonstrated that TPA upregulated Snail at the mRNA and protein levels, increasing its stabilization and its functionality in an ERK1/2 dependent manner.

### 2.4. Snail Is Necessary, but Not Sufficient, for TPA-Induced EMT

**Figure 5:**
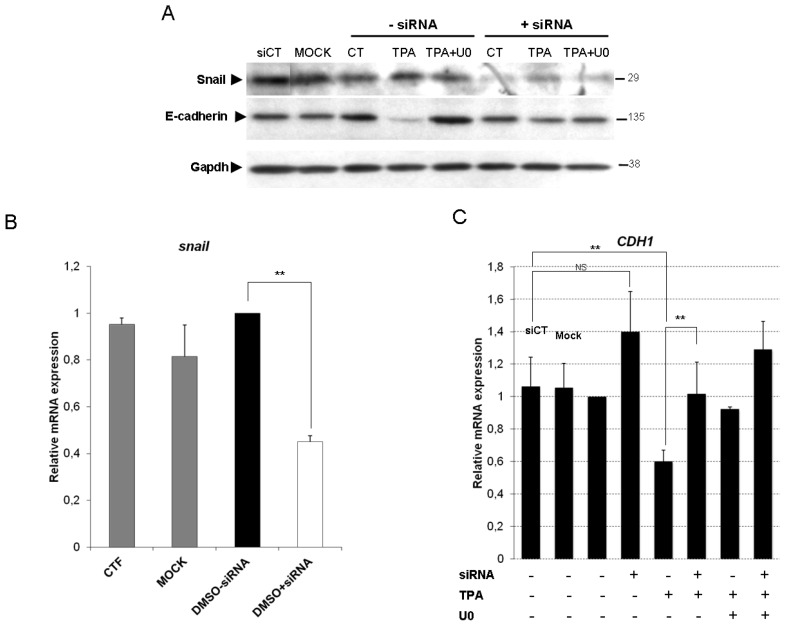
The expression of E-cadherin upon TPA exposure is restored by Snail silencing and ERK1/2 inhibition. HepG2 cells were transfected by incubation with either 40 pmol Snail siRNAs or nonspecific siRNAs (CT) for 36 h. “Mock” indicates that the cells were subjected to transfection conditions in the absence of siRNA. HepG2 siRNA transfectants were exposed to 100 nM TPA, with or without U0126. (**A**) Total proteins were extracted after 48 h and the expression level of Snail and E-cadherin was examined by western blotting. As a control, the same membranes were also probed with an antibody directed against Gapdh. *Snail* (**B**) and *CDH1* (**C**) mRNA levels were assessed by real-time RT-PCR after 48 h of TPA treatment, with or without U0126. Relative mRNA expression levels (normalized with respect to *gapdh*) were determined and mRNA levels in DMSO-treated cells (untransfected condition) were set to 1. Error bars indicate the means ± SEM of triplicate determinations from three independent experiments. ** *p <* 0.01.

We used siRNAs to knock down endogenous Snail expression in HepG2 cells, to confirm the essential role of Snail in TPA-driven EMT. We first assessed the ability of Snail siRNAs to knock down Snail expression. The transfection of cells with Snail siRNA significantly decreased Snail protein (≈75%) and mRNA (≈60%) levels (Figure 5A,B, respectively).

**Figure 6:**
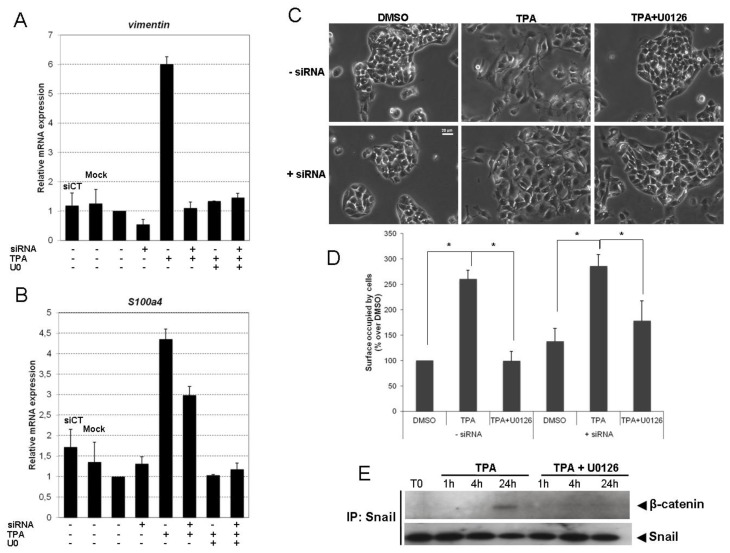
Snail knockdown restores the TPA-induced EMT only partially. (**A**–**C**) HepG2 cells were transfected by incubation with either 40 pmol Snail siRNAs or nonspecific siRNAs (CT) for 36 h. HepG2 siRNA transfectants were exposed to 100 nM TPA, with or without U0126. *Vimentin* (**A**) and *S100a4* (**B**) mRNA levels were assessed by real-time RT-PCR after 48 h of TPA treatment, with or without U0126. Relative mRNA expression levels (normalized with respect to *gapdh*) were determined and mRNA levels in DMSO-treated cells (untransfected condition) were set to 1. Error bars indicate the means ± SEM of triplicate determinations from three independent experiments; (**C**) 48 h after TPA treatment with or without U0126, cell morphology was examined under a light microscope; (**D**) Surfaces occupied by cells were quantified using Nikon software after acquisition of photography using the CCD camera coupled to inverted microscope. Results are expressed as percentage over DMSO (untransfected condition), designated as 100%. Error bars indicate the means ± SEM of triplicate determinations from two independent experiments. * *p <* 0.05; (**E**) The complex of Snail/β-catenin was examined by co-immunoprecipitation assays at 1, 4 or 24 h on HepG2 exposed to TPA with or without U0126. The co-immunoprecipitation results are representative of three independent repeats for each experiment.

Snail silencing resulted in significantly lower levels of TPA-induced E-cadherin protein and mRNA loss (Figure 5A,C, respectively) than observed after transfection with nonspecific siRNA (siCT) and in mock transfection conditions. Having confirmed the essential role of Snail in TPA-driven E-cadherin repression, we investigated the contribution of Snail and ERK1/2 signaling pathways to the EMT of TPA-exposed HepG2 cells. The induction of vimentin (Figure 6A) and fibronectin (data not shown) by TPA was prevented by both Snail siRNA and ERK1/2 inhibition. TPA-induced S100a4 expression was partially restored by Snail siRNA, and totally repressed by U0126 (Figure 6B). These results are consistent with the poor efficacy of Snail silencing against EMT morphological changes in HepG2 cells exposed to TPA (Figure 6C). Indeed, the morphological modifications induced by TPA were strongly inhibited by exposure to U0126 (Figure 6C, TPA + U0126, −siRNA), whereas Snail siRNA was less effective at preventing development of the spindle-shaped fibroblast-like cell morphology (Figure 6C, TPA, +siRNA). As shown in Figure 6E, the area occupied by cells in TPA condition is almost the same whether it is with or without siRNA (Figure 6D), suggesting that Snail silencing has only a slight effect on cell spreading induced by TPA.

S100a4 is a known Wnt target gene, suggesting a possible role for TPA in the activation of β-catenin/TCF signaling. Moreover, it has been shown that Snail can increase Wnt-dependent target gene expression by interacting functionally with β-catenin. We therefore investigated whether Snail associated with β-catenin upon TPA treatment. Co-immunoprecipitation and western blotting demonstrated that Snail bound to β-catenin (Figure 6E). This binding was enhanced at 24 h of TPA treatment and was significantly inhibited by U0126.

These results suggest that there is cross-talk between the Snail and β-catenin signaling pathways in the commitment of EMT induced by TPA in HepG2 cells.

### 2.5. TPA Activates the Wnt/β-Catenin Signaling Pathway

**Figure 7:**
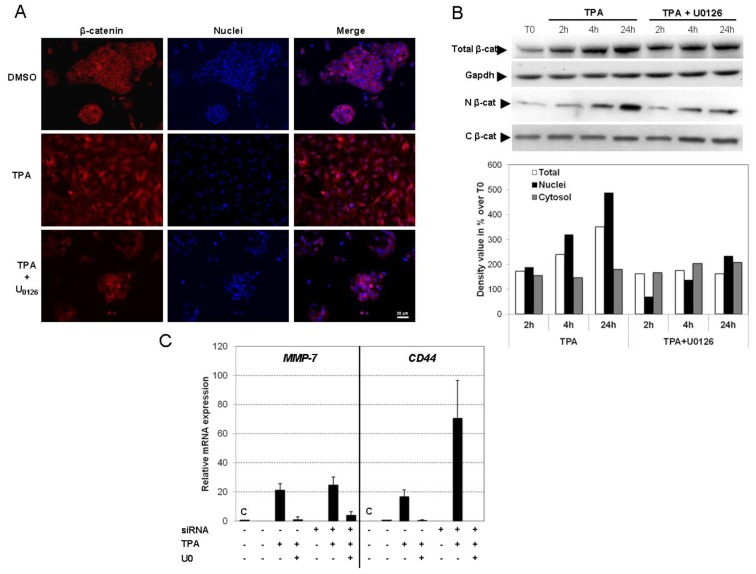
TPA promotes EMT through concomitant activation of the Snaill and Wnt/β-catenin pathways. (**A**) HepG2 cells were grown on coverslips and treated with TPA with or without U0126 for 48 h. After exposure, the cells were fixed and processed for indirect immunofluorescence analysis for the detection of β-catenin (red) and visualization of nuclei (DAPI, blue); (**B**) At indicated time, nuclear (N) and cytosolic (C) fractions were prepared as described in materials and methods, and β-catenin was detected by Western blotting. The semi-quantification of chemiluminescence was performed after the acquisition with a CCD camera. Results are expressed as a percentage of T0-treated cells, designated as 100%. The results are representative of three independent repeats for each experiment; (**C**) Changes in mRNA levels for *MMP-7* and *CD44* genes upon TPA treatment were assessed by real-time RT-PCR after transfection of control siRNA (C) or Snail siRNA. The HepG2 cells were stimulated with 100 nM TPA, with or without U0126 for 48 h. Relative mRNA expression levels (normalized with respect to *gapdh*) were determined and mRNA levels in DMSO-treated cells (untransfected condition) were set to 1. Error bars indicate the means ± SEM of triplicate determinations from three independent experiments.

β-catenin signaling requires stabilization of the cytoplasmic component of β-catenin, which then enters in nucleus, where it associates with and activates TCF/LEF transcription factors. We therefore investigated whether the EMT process observed in HepG2 cells exposed to TPA resulted from the co-activation of Snail and the Wnt/β-catenin pathway. The distribution of β-catenin within the cell was analyzed by western blotting (Figure 7A) and immunofluorescence analysis (Figure 7B) in TPA-treated HepG2 cells. Total β-catenin levels increased, in a time-dependent manner, in cells exposed to TPA (Figure 7A), without mRNA induction (data not shown). These findings are consistent with stabilization of the β-catenin protein. A significant increase in the amount of β-catenin in the nucleus of the cells was observed within 4 h of incubation with TPA, whereas cytoplasmic protein levels remained unchanged. This nuclear translocation was reversed by U0126. For confirmation of these results, we visualized the distribution of β-catenin, by immunofluorescence imaging. As expected, in basal conditions, HepG2 cells showed staining of the plasma membrane and weaker staining of the nucleus. These cells are known to co-express the wild-type and a constitutive active truncated mutant of β-catenin, resulting in a dual distribution, at the membrane and in the nucleus. However, in the presence of TPA, marked nuclear staining of endogenous β-catenin was observed (24 h), this effect being prevented by U0126 (Figure 7B).

As nuclear β-catenin is the hallmark of active Wnt signaling, we investigated TCF/LEF activation, by assessing expression of the *MMP-7* and *CD44* genes by qPCR, two known down-stream target genes of the β-catenin/TCF transcription complex. A large increase in *MMP-7* and *CD44* gene expression was observed in HepG2 cells exposed to TPA (Figure 7C). Interestingly, U0126 prevented this induction of gene expression, whereas Snail siRNA had no such effect. These findings suggested a Snail-independent regulation of these critical genes.

Thus, TPA induces ERK1/2-dependent β-catenin translocation into the nucleus, leading to the activation of specific target genes dependently and independently of Snail activation. These data indicate that TPA-driven EMT requires both the Snail and β-catenin activation pathways and is dependent on ERK1/2.

### 2.6. Discussion

Features of EMT have been observed in cancers developing in several tissues. In the liver, there is a body of evidence to suggest that hepatocellular EMT plays a key role in the dissemination of malignant hepatocytes during HCC progression, allowing hepatic tumor cells to invade the capsule or the portal vein [9]. EMT is now recognized as key process in the mechanisms of metastasis, but the molecular signaling processes underlying EMT in HCC remain poorly understood. This study is the first to reveal the complex networks implicated in EMT in HCC, involving crosstalk between Snail and the β-catenin/Wnt pathway, governed by ERK1/2.

Our results highlight the importance of ERK signaling in the onset of hepatocellular EMT in response to TPA stimulation. We found that the inhibition of ERK1/2 signaling prevented E-cadherin repression, mesenchymal marker induction, cell scattering and the acquisition of a spindle cell-like phenotype during EMT. A crucial role for ERK1/2 signaling pathways in the onset and progression of EMT in several cell or biological models has been widely demonstrated [11,34,35]. Previous experimental studies have shown that the ERK pathway is necessary either for mesodermal gene induction during development [36,37] or for the full EMT induced by several stimuli in tumor cell lines [38–41]. For instance, it has been shown that U0126 completely abolishes the mesenchymal conversion of the thyroid epithelial cells in response to costimulation with TGF-β1 and EGF [39]. Levels of ERK1/2 production and activity are significantly higher in HCC models and human HCC tissue specimens than in healthy liver [42,43]. Moreover, MAPK/ERK activity has been shown to be positively correlated with tumor size and aggressive tumor behavior [44,45], suggesting that ERK1/2 activation reflects aggressive tumor behavior in clinical conditions.

Our findings, together with those of previous studies, provide evidence for a crucial role of Snail in E-cadherin repression by TPA. Moreover, we show that the transcription of Snail and the binding of this factor to the *CDH1* promoter following exposure to TPA are dependent to the activation of ERK1/2. These observations were supported by the inhibition of the Snail induction and activation (qPCR and ChIP experminents, respectively) by U0126, and are also consistent with previous findings. For example, it has been shown that suppression of ERK1/2 activity in MCF7 or HEK293 cells correlated with the downregulation of Snail [33]. Moreover, the ERK pathway leads to the activation of the transcription factor Snail1 in chick embryo that undergoes a process of EMT in the central dermomyotome [46]. We notably found that ERK1/2 was involved in the binding of Snail to the *CDH1* promoter and, thus, in the repression of E-cadherin gene transcription.

Snail activity can also be regulated post-translationally [43], but Snail gene transcription generally displays the reverse pattern to E-cadherin gene transcription, being detected in cells that have lost their epithelial characteristics. Indeed, an inverse correlation between E-cadherin and Snail levels has been reported in a panel of epithelial and dedifferentiated cells derived from carcinomas of various types, including HCC [47]. EMT is initiated principally by the abolition of E-cadherin gene expression by zinc-finger proteins (e.g., Snail, Slug) or bHLH family transcriptional factors (e.g., Twist), which bind to three E-boxes located in the proximal promoter of *CDH1* [48]. The disruption of E-cadherin-mediated adhesion is thought to be a key step in progression toward the invasive phase of hepatocarcinoma [49–51]. The connection between the loss of E-cadherin function in cancer cells and the occurrence of EMT is well documented. E-cadherin downregulation in HCC is associated with increases in tumor size, low levels of histological differentiation, invasion recurrence, metastasis and poor prognosis [19,52]. The disruption of E-cadherin/β-catenin complexes at cell-to-cell junctions is characteristic of hepatocellular EMT. Indeed, E-cadherin is frequently found in the cytoplasm and may even be entirely absent in poorly differentiated HCC, as it remains at the plasma membrane in well-differentiated human HCC [9,20,53]. Increases in the expression of E-cadherin repressors, such as Snail, constitute a critical step in EMT in HCC, as demonstrated by the acceleration of invasion by Snail overexpression [54,55].

Furthermore, in addition to its role in the repression of E-cadherin, Snail is also known to stimulate mesenchymal gene transcription [56]. We found that TPA induced the production of vimentin and fibronectin and that is this induction was dependent on Snail and ERK1/2. However, we demonstrated that Snail binding to and repression of the E-cadherin gene was not sufficient to induce a complete morphological transformation, unlike exposure to U0126. Indeed, Snail knockdown had only a limited effect on the induction by TPA of a spindle-shaped fibroblast-like morphology. Thus, Snail is not exclusively responsible for full commitment to the EMT process induced by TPA. It has been shown that, in some instances, Snail could induce an incomplete EMT. For instance, in mice lacking Snail, the cells of the primitive streak begin to invade, however the EMT is incomplete [57]. Moreover, a study demonstrated that hypoxia condition could induce partial EMT of breast cancer cells where induction of Snail does not elicit a motile phenotype [58]. By analogy, a loss of E-cadherin has been shown to be necessary, but not always sufficient, for the induction of full EMT [59]. For example, the ectopic expression of E-cadherin does not restore the epithelial phenotype in cells overproducing Twist [29,60]. Taken together, our findings imply that TPA-induced EMT involves another signaling pathway in addition to Snail activation.

Although Snail plays a critical role in EMT, the repression of E-cadherin gene transcription has frequently been reported to occur in tandem with activation of the Wnt signaling cascade [61,62]. Wnt signaling may therefore help to stabilize the pool of β-catenin released after Snail-mediated E-cadherin repression [63,64]. Moreover, direct interactions between the Wnt signaling pathway and Snail activation have been demonstrated. Indeed, Stemmer *et al.* showed that Snail can interact physically and functionally with β-catenin, to increase Wnt-dependent target gene expression [65]. This association is thought to stimulate the Wnt pathway in a positive feedback loop. Consistent with this view, we found that TPA-induced ERK1/2 activation led to a physical interaction between Snail and β-catenin in differentiated hepatoma cells.

The Wnt/β-catenin signaling pathway plays a key role in liver development, growth, regeneration, zonation, metabolism and oxidative stress [66]. It is also involved in the development of various liver diseases, ranging from hepatitis to HCC. Indeed, abnormal regulation of the transcription factor β-catenin has been identified as a major, early carcinogenic event in HCC development [67]. Intriguingly, we found that ERK1/2, but not Snail, was required for the induction of MMP-7 and CD44. These results are consistent with an activation of the Wnt pathway that is dependent on ERK1/2 activation but independent of Snail. It thus seems likely that TPA-triggered ERK1/2-dependent EMT may result from the combined activation of the Snail/β-catenin axis and the Wnt pathway.

Aberrant activation of the Wnt/β-catenin pathway has been observed in 18%–67% of HCC tumors and has been shown to play an important role in hepatocarcinogenesis [68]. β-catenin mutations have been identified as chief activators of the Wnt pathway in HCC. Such alterations were therefore believed to be involved in the occurrence and development of HCC [69–71]. We used the HepG2 cell line, a well-differentiated cell line derived from human HCC. These cells produce both full-length (wild-type) and a truncated form of β-catenin lacking the phosphorylation sites for GSK3 [72]. Our findings are consistent with previous reports of a dual membrane and nuclear distribution of the β-catenin protein [73]. We found that, despite the constitutive activation of canonical Wnt signaling in HepG2 cells, TPA increased the nuclear translocation of β-catenin and activation of the Wnt signaling pathway. We found that the stabilization of β-catenin in the nucleus after TPA exposure was associated with the overproduction of MMP-7 and CD44, two proteins directly involved in invasion and metastasis [74,75].

## 3. Experimental Section

### 3.1. Materials

Dulbecco’s modified Eagle’s medium and fetal bovine serum (FBS) were obtained from Invitrogen (Rockville, MD, USA) and penicillin/streptomycin solution was obtained from Bio-Whittaker (CAMBREX Company, Walkersville, MD, USA). U0126 was purchased from Cell Signaling (Danvers, MA, USA). All other chemicals were obtained from Sigma-Aldrich (L’Isle d’Abeau Chesne, Saint Quentin Fallavier, France) unless otherwise specified. The antibodies specific for pERK1/2, pMEK1/2, ERK2, pP90RSK, pBad and Gapdh (Cell Signaling Technology, Danvers, MA, USA), E-cadherin, fibronectin (Epitomics, Burlingame, CA, USA), β-catenin and Snail (Santa Cruz, Heidelberg, Germany) were used for western blotting and immunodetection experiments (Table 1).

### 3.2. Cell Culture and Drug Treatments

HepG2 was obtained from ATCC (American Type Culture Collection, Manassas, VA, USA) and maintained in DMEM supplemented with 10% FBS and 1% penicillin/streptomycin at 37 °C, under a humidified atmosphere containing 95% air and 5% CO_2_. For all the experiments, the FBS content of the medium was decreased to 5%. TPA was added to the culture medium at a concentration of 100 nM for the times indicated. The mitogen-activated protein kinase kinase (MEK)1/2 inhibitor U0126 was added before TPA treatment, at a concentration of 5 μM. U0126 and TPA were prepared as stock solutions in dimethylsulfoxide (DMSO). The final concentration of DMSO in the medium was 0.25% in all conditions.

### 3.3. Western Blot Analysis

Western blot analysis was performed as previously described [76]. Target protein levels were determined by immunoblotting with the corresponding primary antibodies for 1 h and then incubating the membrane with horseradish peroxidase-conjugated secondary antibodies (anti-mouse immunoglobulin G or anti-rabbit immunoglobulin G; Promega, Madison, WI, USA). Signals were detected with Immobilon Western Detection Reagents (Millipore, Molsheim, France) and acquired with a CCD camera (ChemiGenius2, SynGene, Sunnyvale, CA, USA). Semi-quantitative analysis was then carried out with GeneTools software.

### 3.4. Immunofluorescence Staining

Hepatoma cells seeded on glass coverslips in 12-well plates (4 × 10^5^ cells per well). After drug exposure, the cells were fixed with 4% paraformaldehyde, permeabilized with 0.5% saponin and incubated with the corresponding primary antibodies for 1 h. They were then probed by incubation with goat anti-rabbit or anti-mouse IgG coupled to AlexaFluor^®^ 488 or 594 (Molecular Probes, Eugen, OR, USA) for 1 h. Nuclei and F-actin were stained with 2,6-diamidino-2 phenylindole (DAPI) and AlexaFluor 488-conjugated phalloidin (Molecular Probes, Eugen, OR, USA), respectively. Slides were mounted and sealed in ProlongGold antifade reagent (Invitrogen, Rockville, IN, USA). Images were acquired with an inverted fluorescence microscope (Nikon, Amstelveen, The Netherlands) equipped with a CCD camera (ORCA ER, Hamamatsu Photonics, Massy, France), at 20× magnification.

### 3.5. Cell Migration Assay

HepG2 cells were used to seed six-well plates (2 × 10^6^ cells per well) and were cultured until they formed a monolayer (~48 h later). Crossing wounds were created with a sterile pipette tip. The cell migration progress was photographed in three regions immediately (T0) and 24 h after treatments, with an inverted microscope (Nikon) equipped with a CCD camera (ORCA ER, Hamamatsu Photonics), at ×4 magnification. Automated analyses were carried out with Tscratch software [77].

### 3.6. Reverse Transcription-Quantitative Polymerase Chain Réaction

RNAs were isolated and cDNA synthesized as previously described [76]. Quantitative PCR analysis was carried out with LightCycler^®^480 Probes Master (Roche Applied Science, Meylan, France), according to the manufacturer’s instructions, together with FAM-labeled hydrolysis probes from the Universal Human Probe Library Set (Roche). Intron-spanning primers were designed with Universal Probe Library Assay Design Center software (https://www.roche-applied-science.com/sis/rtpcr/upl/index.jsp?id=uplct_030000). Calculations were carried out with *gapdh* as the endogenous control reference gene. Fold differences in gene expression were calculated with LightCycler software, taking into account the efficiency of amplification, determined from a standard curve obtained with the second-derivative maximum method.

### 3.7. Chromatin Immunoprecipitation

Chromatin immunoprecipitation (ChIP) assays were performed with the EZ-ChIP Kit (Upstate Biotechnology, Millipore, Molsheim, France), according to the manufacturer’s instructions. Briefly, immunoprecipitation was carried out with specific antibodies directed against Snail (Santa Cruz, Heidelberg, Germany), normal rabbit IgG (as a negative control, Millipore, Molsheim, France) or against RNA Pol II (as a positive control, mouse monoclonal IgG1, Millipore, Molsheim, France) for 16 h at 4 °C, with rotation. The purified immunoprecipitated DNA was eluted in 20 μL. ChIP DNA was analyzed by PCR, with specific primers amplifying two fragments of the *CDH1* promoter and one fragment of the 3′-untranslated region (UTR), as described by Saito *et al.* [78]. The amplified DNA was subjected to electrophoresis in a 2% agarose gel and visualized by ethidium bromide staining. The binding of Snail to DNA was assessed by calculating dividing the intensity for each bound sample by the input, dividing the value for the vehicle control by the input and then calculating the ratio of the two results.

### 3.8. Small Interfering RNA (siRNA)

HepG2 cells were used to seed 12-well plates (1.2 × 10^5^) and were transfected with 40 pmol control, Snail siRNA pool (Santa Cruz) or ERK1/2 siRNA (Santa Cruz), in the presence of interferin transfection reagent (Polyplus-Transfection, Illkirch, France), according to the manufacturer’s instructions. After 24 h of incubation at 37 °C, cells were treated with TPA, with or without U0126. At each of the times indicated, RNA and proteins were extracted for quantitative PCR and western blotting analysis, respectively.

### 3.9. Statistical Analysis

All experiments were carried out at least three times. Data are expressed as means ± standard deviations (SD). The statistical significance of differences between samples was determined in Student’s *t* test. Probability is indicated as follows: *p < 0.05 or **p < 0.01.

## 4. Conclusions

**Scheme 1:**
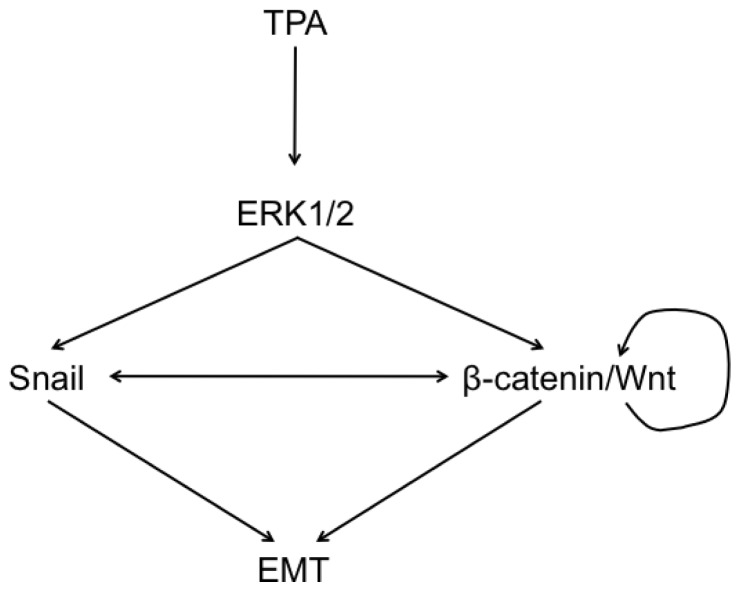
A simplified summary of the signaling involved in response to TPA and leading to the process of EMT on HepG2 cells. This study highlights the formation of a complex network governed by ERK1/2 signaling pathway, converging on the coregulation of Snail and the Wnt/β-catenin pathway and responsible for the onset and the progression of EMT in hepatocellular carcinoma cells.

This study reveals the key role of ERK1/2 in an EMT process regulated by Snail and the Wnt/β-catenin signaling pathway, implying a balanced mechanism in the dedifferentiation of hepatocellular liver carcinoma cells (Scheme 1).

Our work sheds new light on mechanisms that could be targeted in new molecular approaches. Identification of the genetic controls and biochemical mechanisms underlying acquisition of the invasive phenotype and the subsequent spread of hepatocellular cells is of prime importance when selecting potential treatment targets. Inhibition of the ERK pathway can reduce, or even entirely abolish EMT in the liver. These findings imply that the targeting of ERK signaling in HCC could potentially improve outcomes, restricting the progression of this disease.

## Figures and Tables

**Table 1:** Primary antibodies used for Western blot and Immunofluorescence staining.

Antigen	Phosphorylation site	Source/type	Manufacturer	Dilution WB	Dilution IF
pErk1/2	Thr202/Tyr204	Rabbit mAb^1^	Cell signaling	1:2000	
pErk1/2	Thr202/Tyr204	Mouse mAb	Sigma		1:300
pMEK1/2	Ser217/221	Rabbit pAb^2^	Cell signaling	1:2000	
Erk2		Rabbit pAb	Cell signaling	1:5000	
pP90^RSK^	Ser380	Rabbit pAb	Cell signaling	1:2000	
pBad1	Ser136	Rabbit pAb	Cell signaling	1:2000	
Gapdh		Rabbit mAb	Cell signaling	1:7500	
E-cadherin		Rabbit mAb	Epitomics	1:5000	
b-catenin		Rabbit mAb	Santa Cruz	1:2000	1:300
Fibronectin		Rabbit mAb	Epitomics	1:1000	1:400
Snail		Rabbit pAb	Santa Cruz	1:1000	

1 mAb, monoclonal antibody;

2 pAb, polyclonal antibody.
